# A Comprehensive Review of Margin Identification Methods in Soft Tissue Sarcoma

**DOI:** 10.3390/curroncol32120703

**Published:** 2025-12-13

**Authors:** Yasmin Osman, Jean-Philippe Dulude, Frédéric Leblond, Mai-Kim Gervais

**Affiliations:** 1 Division of General Surgery, Department of Surgery, Universite de Montreal, Montreal, QC H3T 1J4, Canada; yasmin.essam.osman@umontreal.ca (Y.O.); jean-philippe.dulude@umontreal.ca (J.-P.D.); 2 Department of Physics Engineering, Polytechnique, Montreal, QC H3T 0A3, Canada; frederic.leblond@polymtl.ca; 3 Centre de Recherche du Centre Hospitalier de l’Universite de Montreal (CHUM), Montreal, QC H2X 0C1, Canada; 4 Division of Surgical Oncology, Department of Surgery, Maisonneuve-Rosemont Hospital, Montreal, QC H1T 2M4, Canada

**Keywords:** soft tissue sarcoma, margin assessment, intraoperative imaging, fluorescence-guided surgery

## Abstract

Soft tissue sarcomas (STS) are rare and heterogeneous tumors for which achieving complete tumor resection with negative surgical margins remains the cornerstone of curative treatment and a key predictor of survival. Current intraoperative resection margin status assessment techniques remain limited, as traditional intraoperative frozen section analysis is of limited accuracy for most STS histological subtypes. This comprehensive review evaluates current and emerging margin assessment techniques used intra-operatively during STS resection. A systematic search of PubMed and PubMed Central databases from 2000 to 2025 identified studies using fluorescence imaging, spectroscopy, and ultrasound-based modalities. Indocyanine green (ICG) fluorescence-guided surgery appeared to be the closest to widespread use, with the most clinical evidence showing potential to reduce positive margins. Use of acridine orange (AO) as a fluorescent dye also showed potential in decreasing local recurrences, but it remains in the experimental stage of research with little clinical data available. Raman spectroscopy has recently shown high accuracy in identifying STS from healthy tissue, but the impact of its use on patient outcomes has not been studied yet. Other techniques, such as diffuse reflectance spectroscopy (DRS), rapid evaporative ionization mass spectrometry (REIMS), optical coherence tomography (OCT), and intraoperative ultrasound (IOUS) yielded encouraging results but still require further prospective studies to validate their safety, reproducibility, and clinical utility in improving surgical precision and patient outcomes.

## Simple Summary

Soft tissue sarcomas (STS) are rare and heterogeneous tumors for which achieving complete tumor resection with negative surgical margins remains the cornerstone of curative treatment and a key predictor of survival. This review presents emerging technologies designed to improve real-time margin assessment, including fluorescence-guided imaging with indocyanine green (ICG), acridine orange (AO), Raman spectroscopy, diffuse reflectance spectroscopy (DRS), rapid evaporative ionization mass spectrometry (REIMS), optical coherence tomography (OCT), and intraoperative ultrasound (IOUS). Each technique offers distinct benefits while facing limitations in sensitivity and feasibility. Among these methods, ICG demonstrates the most clinical evidence for reducing positive margin prevalence along with safety and applicability, while others remain at early investigational stages. Continued research is needed to obtain further clinical validation and clarify their roles in improving intraoperative precision and oncologic outcomes in STS surgery.

## 1. Introduction

Soft tissue sarcomas (STS) are a rare and heterogeneous group of mesenchymal malignancies, accounting for less than 1% of all newly diagnosed cancers annually [1]. Approximately 13,000 new cases are diagnosed yearly in the United States, and 1700 cases in Canada [2,3]. In population-based analyses, the crude national incidence in Canada has been estimated at approximately 43.8 cases per million individuals annually, consistent with the international incidence range of 40–50 cases per million [4]. Despite their rarity, the global burden of sarcoma—including incidence, prevalence, mortality, and disability-adjusted life-years (DALYs)—has increased by 42.8% over the past two decades [5]. These tumors can present with significant challenges in surgical management, and their treatments must be tailored according to their histological subtypes, anatomical location, and biological behavior [6,7,8]. The most common site for STS remains the extremities, and less frequently the trunk and chest wall, head and neck, and retroperitoneum [2,3,4,5,6,7,8,9]. The most common histological subtypes in the retroperitoneum include liposarcomas, leiomyosarcomas, and undifferentiated pleomorphic sarcomas [10].

Surgery remains the cornerstone of curative treatment, with en-bloc resection (comprehensive surgery) and wide surgical margins considered the standard approach [11,12,13]. The primary goal of STS surgery is to achieve complete resection, to improve survival, and to minimize the risk of recurrence [14,15]. Historically, the five-year local recurrence (LR) following complete retroperitoneal sarcoma (RPS) resection was 50% [16,17]. The implementation of compartmental (en bloc) resection techniques has reduced local recurrence to less than 30% [18,19]. Extremity and trunk wall sarcomas have a reported recurrence risk of approximately 11–12% at ten years [20,21].

In STS, local recurrence represents a predictor of overall survival (OS) [22]. The risk of recurrence can be influenced by several factors, including tumor localization, histological subtype, grade, age, tumor size, and surgical margins status [23,24]. In fact, studies have demonstrated that positive surgical margins are associated with recurrence risks that can exceed 50% [25,26,27]. In a study including 347 patients undergoing surgery for non-metastatic primary RPS, patients undergoing R2 resections (gross residual disease) have a median survival of only 18 months, compared with >100 months for R0 (negative microscopic margins) resections [28]. However, this prognostic effect is not uniform across subtypes. Well-differentiated liposarcoma shows no clear survival disadvantage with positive margins, unlike higher-grade or dedifferentiated liposarcoma [29,30]. Despite the importance of achieving negative margins, there is no consensus on the definition of an adequate negative margin. The American Joint Committee on Cancer (AJCC) classifies margins as R0 (negative), R1 (microscopically positive), and R2 (grossly positive), yet studies have reported a wide range of recommended margin distances, from <1 mm to >10 mm, mostly depending on histological subtypes [25,31].

According to the National Comprehensive Cancer Network guidelines, “oncologically appropriate margins” imply pathologically negative resection margins [14]. Planned close margins or microscopically positive margins (R1) may be adequate depending on tumor subtypes, with the objective of sparing critical structures and preserving function [31]. Beyond margin width, the concept of margin “quality”—defined by the presence of natural tissue barriers such as fascia, periosteum, peritoneal surface, and vascular adventitia—is increasingly recognized as a critical factor in recurrence risk [25,26,27,28,29].

Given the importance of margin status and completeness of resection, there is growing interest in the integration of intraoperative imaging and margin assessment technologies to improve real-time tumor identification and resection precision in STS. Emerging techniques such as fluorescence-guided surgery have shown potential for improving the visualization of tumor margins in sarcoma and other tumors [32,33,34,35,36,37]. Optical coherence tomography (OCT) has been proven to differentiate tumor from normal tissue at micrometric resolution [38]. Additionally, Raman spectroscopy has been studied for use in rapid, real-time tumor delineation [39,40,41]. Although multiple studies present promising results, there exists no consensus on the comparison of their accuracy, clinical utility, and impact on oncologic outcomes in STS.

There remains a clear gap in the literature regarding the comparative performance and clinical applicability of emerging intraoperative margin-assessment technologies in soft tissue sarcoma (STS) surgery. To address this, our review synthesizes the current evidence on real-time imaging modalities—including fluorescence-guided surgery, optical coherence tomography, and Raman-based techniques—by evaluating their diagnostic performance, technical feasibility, and stage of clinical validation.

The primary objective of this review is to present the various emerging intraoperative margin assessment methods in STS surgery, as well as their advantages and limitations. Secondary outcomes of our study are to evaluate the potential impact on oncologic outcomes and the improvement of surgical precision.

## 2. Materials and Methods

We conducted a structured evidence review using STROBE guidelines [42]. A systematic search of PubMed and PubMed Central was performed for articles published between 1 January 2000 and 31 December 2025. The search included the following key terms: “soft tissue sarcoma,” “margin assessment,” “intraoperative imaging,” “frozen section,” “fluorescence-guided surgery,” “Raman spectroscopy,” “optical coherence tomography,” and “ultrasound.” Studies published in English between 2000 and 2025 were included, with priority given to prospective trials, meta-analyses, and retrospective studies evaluating margin identification techniques.

Eligible studies were selected based on the following inclusion criteria: (1) studies reporting original outcomes related to intraoperative margin assessment in STS surgery; (2) studies evaluating imaging modalities for margin identification relevant to STS surgery; (3) studies investigating novel margin assessment techniques, including fluorescence imaging, spectroscopy, and artificial intelligence-based approaches.

The exclusion criteria included the following: (1) preoperative imaging only (MRI/CT/PET); (2) non-sarcoma populations without STS subgroup data; (3) review articles, editorials, case reports, and conference abstracts. A total of 178 publications were identified. After the removal of duplicates, 150 unique articles were screened by title and abstract by two coauthors (YO and JPD). Of these, 44 full-text articles were evaluated for eligibility.

**Figure 1:**
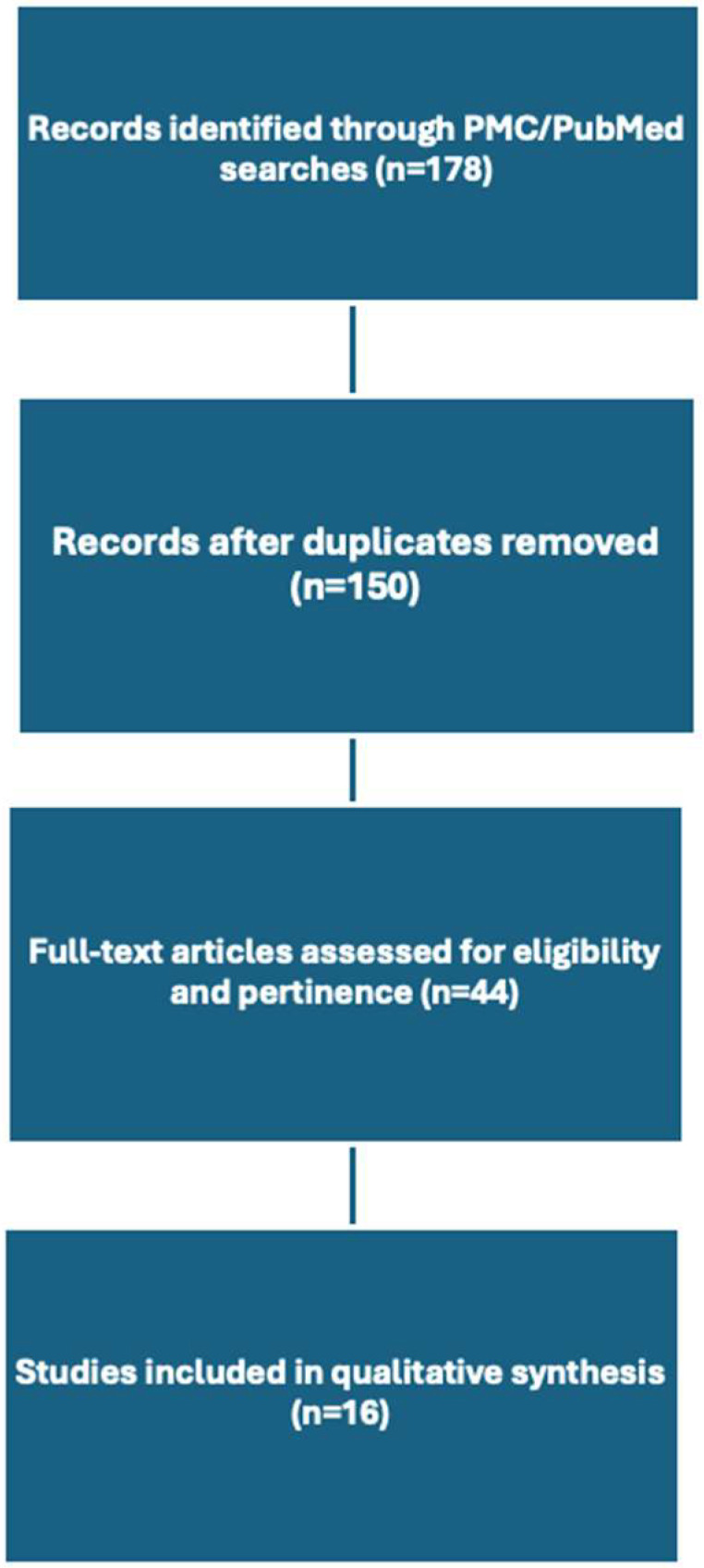
STROBE flow diagram.

A total of 16 studies met the inclusion criteria and were included in the qualitative synthesis. A STROBE flow diagram summarizing the selection process is provided in Figure 1. Studies were grouped by modality: indocyanine green fluorescence, acridine orange, Raman spectroscopy, diffuse reflectance spectroscopy, rapid evaporative ionization mass spectrometry, optical coherence tomography, and intraoperative ultrasound.

## 3. Results: Margin Assessment Techniques

### 3.1. Frozen Section and Histopathology

Frozen section analysis is an intraoperative histopathological technique in which fresh tissue is rapidly frozen, thinly sectioned, stained, and immediately examined microscopically by a pathologist to provide real-time diagnostic information [43].

Limited evidence exists for the routine use of intraoperative frozen section in STS. Even if this technique is the most universally used intraoperatively, studies have shown that it does not reliably predict final pathology and can be falsely reassuring [44]. It is also associated with added cost and minimal impact on surgical decision-making [44]. In a study including 179 patients treated for STS of the trunk and extremities from 2005 to 2019, 66% had peripheral margins sampled, and 8.4% had positive margins on final pathology. Only one intraoperative frozen margin led to additional per-operative resection, and three patients with R1 resections had false-negative frozen peripheral margins [44].

While histopathologic assessment continues to be the gold standard for final margin evaluation, frozen section does not consistently improve surgical outcomes or reduce local recurrence in STS [25,26,27,28,29,30,31,32,33,34,35,36,37,38,39,40,41,42,43,44,45].

### 3.2. Fluorescence-Guided Surgery

#### 3.2.1. Indocyanine Green

The use of indocyanine green (ICG) fluorescence dye imaging has emerged as a promising adjunct for intraoperative tumor visualization. ICG binds plasma proteins and accumulates in tumors due to clarithrin-mediated endocytosis and the enhanced permeability and retention (EPR) effect, providing real-time surgical guidance [35,36,37,38,39,40,41,42,43,44,46]. It fluoresces in the near-infrared (NIR) spectrum and has an excellent safety profile [33]. Beyond sarcoma, ICG-guided margin assessment has been explored in multiple malignancies, including breast cancer, gastric cancer, primary liver tumors such as hepatocellular carcinoma, colorectal liver metastases, primary lung cancers and pulmonary metastases, and sentinel lymph node mapping [32,36,47,48].

In STS, Nicoli et al. evaluated the feasibility and drawbacks of near-infrared fluorescence imaging with preoperative indocyanine green (75 mg; within the recommended 2 mg/kg limit). The cohort mainly included extremity sarcomas (8/11), with additional cases located in the groin, chest wall, and pelvis. Tumor fluorescence was observed in 9 of 11 cases (81%). The two non-fluorescent tumors included an osteosarcoma with >90% necrosis and a grade 1 myxofibrosarcoma. No adverse events were reported with the use of ICG. Microscopically positive margins (R1) occurred in two cases, requiring re-excisions. In three cases, intraoperative fluorescence led the surgeon to resect additional tissue [37]. In a larger prospective clinical study, Brookes et al. (2021) evaluated ICG use for 39 high-grade sarcoma resections, including 26 STS. Tumors were primarily located in the lower extremity (*n* = 21), followed by the upper extremity (*n* = 9), pelvis (*n* = 6), trunk (*n* = 2), and groin (*n* = 1). Their study found that ICG use significantly reduced unexpected positive margins (5.1% vs. 25%, *p* = 0.01) [49].

Similarly, in Gong’s study evaluating 18 STS resections, ICG margins correlated with the final pathology in 56% of cases, demonstrating high specificity (89%) but low sensitivity (22.2%) [50]. The surgeon’s impression aligned with pathology in the same proportion of cases. Of six discrepancies between ICG and surgeon assessments, two ICG-positive cases had true positive margins. These results highlighted ICG’s high specificity but limited sensitivity for margin assessment.

In vivo assessment of ICG fluorescence in sarcoma surgery can provide multiple advantages: real-time visualization, improved detection of microscopic infiltration, and a low toxicity profile. However, the main challenge identified with its use remains background fluorescence assessment and signal significance cutoff. Previously used qualitative assessment can lead to over-resection, variable uptake among sarcoma subtypes, and a lack of consensus in optimal dosing and timing of administration [35]. Recent advances in machine learning classification algorithms provide promising prospects in the development of this field [51,52,53].

#### 3.2.2. Acridine Orange

Acridine orange (AO) is a fluorescent dye that selectively accumulates in STS cells due to their acidic microenvironment, high nucleic acid content, and enhanced glycolysis-driven lysosomal acidity that traps the dye [54]. When activated by blue light or low-dose radiation, AO generates reactive oxygen species that disrupt lysosomal membranes, leading to tumor cell apoptosis [54,55]. This dual photodynamic and radio-dynamic effect enables precise tumor targeting while sparing normal tissue, making AO a promising tool for eliminating microscopic residual disease during surgery [56]. Its selective uptake in sarcoma cells allows for fluorescence-guided identification and the removal of remaining cancer cells while minimizing damage to surrounding healthy tissue [56].

In fact, AO-assisted surgery has been associated with significantly lower local recurrence following marginal resections [56,57,58]. In a comparative study, Tsuchie et al. evaluated 19 patients treated with AO therapy—including photodynamic therapy (PDT), followed by photodynamic surgery, and radio-dynamic therapy (RDT)—against 33 patients who underwent marginal resection alone. The procedure involved applying an AO solution to resected surfaces, using fluorescence-guided curettage under xenon lamp illumination to eliminate microscopic residual tumor cells, followed by PDT and an immediate postoperative 5 Gy radiotherapy session after wound closure. The AO group had a significantly lower local recurrence rate (*p* < 0.05) [56]. In addition to reducing local recurrence, the AO cohort featured worse baseline prognostic factors. Multivariate analysis confirmed that high-grade malignancy and absence of AO were independent predictors of recurrence [56].

Similarly, Matsubara et al. compared AO-assisted marginal resections with conventional wide-margin excisions (2–5 cm) and found comparable outcomes, with recurrence risks of 29% and OS of 68%. In this study, with heterogeneous population groups, the use of AO combined with marginal resection in cases of proximity to important anatomical structures produced comparable short-term oncologic outcomes to standard resection [58]. These findings point towards AO’s potential to facilitate function-preserving surgery while maintaining disease control.

AO-assisted surgery could potentially offer the advantage of function-preserving resections by reducing the need for wide resection while appropriately treating the tumor locally. Its selective uptake minimizes damage to healthy tissue, and clinical studies report no severe short-term side effects [56,57,58]. However, limited clinical validation and no FDA approval, the need for specialized fluorescence imaging equipment not routinely available in operating rooms (unlike widely available ICG near-infrared systems), and safety concerns for its long-term mutagenic and genotoxic potential represent significant hurdles to its short-term clinical implementation [54]. While AO shows strong potential in STS resection extrapolated from its in vitro characteristics, further large-scale clinical studies are needed to validate its long-term safety and efficacy.

### 3.3. Spectroscopy

#### 3.3.1. Raman

Raman spectroscopy is a non-invasive optical analysis technique based on inelastic light scattering detection. Raman spectra provide molecular information from the studied sample and can be used as a fingerprint for distinct types of biological tissue [59]. Distinct spectra arise from differences in protein, lipid, and nucleic acid composition of tissues. Neoplastic transformation alters these molecular compositions, enabling Raman-based cancer detection, as demonstrated across breast, brain, gastric, colon, and melanoma tumors [48,49,50,51,52,53,54,55,56,57,58,59,60,61,62,63,64,65,66,67].

A persistent limitation is long acquisition times and poor signal-to-background ratio (SBR), as weak Raman scattering is often masked by tissue fluorescence and instrument noise [68]. Proof-of-concept of use with STS was demonstrated by Wills et al. in 2009 using ex vivo pediatric sarcoma specimens, achieving 94% classification accuracy, though without comparison to healthy controls [69]. Li et al. extended this to adult sarcomas, using confocal microscopy to acquire ex vivo Raman spectra from three patients and developing convolutional neural network classification algorithms. Reported results included high sensitivity (94.9%) and specificity (88.3%) [40]. However, the included subtypes of STS were not detailed, and acquisition times exceeding 30 min per scanned area limited clinical applicability.

The first intraoperative feasibility study using Raman spectroscopy during STS resection was conducted by Nguyen et al. (2017), who obtained spectra from 42 patients. They reported an overall sensitivity of 59.6% and specificity of 91.6%, which improved to 89.5% and 96.4%, respectively, when well-differentiated liposarcomas (WDLPS) were excluded. The poor performance in WDLPS reflected their close spectral similarity to normal adipose tissue. This is consistent with the challenge involved in posing a histopathological diagnosis of WDLPS, and with their similar macroscopic appearance, which poses a critical challenge during retroperitoneal WDLPS resection [39].

Building on these early efforts, Dulude et al. evaluated a new high-speed handheld probe, the Ultra Probe, during resection of retroperitoneal and extremity STS in 30 patients. Raman spectra were acquired from both healthy tissues in vivo and from resected sarcoma specimens. This dataset was used to develop a machine learning random forest classification algorithm. The Ultra Probe reduced acquisition times, allowing seamless integration into the surgical workflow. Reported results included >90% sensitivity, specificity, and accuracy in differentiating liposarcomas from adipose tissue, and >80% in differentiating non-liposarcoma STS from non-adipose healthy tissue. Notably, performance was similar when comparing WDLPS and healthy adipose tissue, with a sensitivity of 94%, specificity of 95%, and accuracy of 94% [41].

Handheld fiber-optic Raman spectroscopy probes have been explored for intraoperative tissue assessment, showing high diagnostic accuracy in differentiating sarcoma from adjacent fat and muscle [40]. Raman spectroscopy thus offers high biochemical specificity and growing intraoperative feasibility, but requires further technical refinement and prospective validation before routine surgical adoption [41]. As with most STS research, population heterogeneity resulting from the large number of histological subtypes limits generalizability from any model that attempts to include multiple subtypes [39,40,41]. Acquisition times also impose a limit to the area from which Raman spectra can be acquired at a single time, with most handheld probes having a tip diameter less than a millimeter squared. Recent developments in wide-field Raman spectroscopy could change this paradigm [70].

#### 3.3.2. Diffuse Reflectance

Diffuse reflectance spectroscopy (DRS) is another non-invasive, real-time optical technique that is simple to use and does not require contrast agents. It operates by delivering broadband light to tissue through an optical fiber and analyzing the reflected spectrum [71]. The interaction of light with tissue occurs through scattering and absorption—absorption is influenced by the tissue’s chemical composition, while scattering is determined by its subcellular morphology. As a result, the altered diffuse reflectance spectrum provides valuable insights into both the structural and compositional properties of the measured tissue [72,73].

This technology has been explored for tumor characterization and margin assessment in various cancers, including breast, colorectal, and lung malignancies [72,73]. Geldof et al. evaluated the effectiveness of diffuse reflectance spectroscopy (DRS) in distinguishing tumors from healthy tissue in 20 freshly excised sarcoma specimens [74]. Using a handheld probe, their method achieved a classification accuracy of 90%, with a sensitivity of 88% and specificity of 93% when including WDLPS. Their results demonstrated consistent performance across various histological subtypes (e.g., myxofibrosarcoma, WDLPS, leiomyosarcoma) and anatomical locations (extremities, retroperitoneum, trunk) [74]. However, the study is limited by a small sample size and the absence of in vivo validation.

While DRS provides advantages such as large measurement volumes and operational simplicity, it faces limitations, including reduced biochemical specificity and the need for direct tissue contact [75].

#### 3.3.3. Rapid Evaporative Ionization Mass Spectrometry (REIMS)

Rapid evaporation ionization mass spectrometry (REIMS) is a direct mass spectrometry technique that uses electrical current to rapidly heat and evaporate biological samples, generating ionized aerosols that are analyzed by mass spectrometry to produce real-time molecular fingerprints of tissue composition [76]. One study at Maastricht University Medical Center presents the analysis of electrosurgical vapors produced during surgery by REIMS as an innovative method to obtain intraoperative molecular information about tissue pathology [77].

In their study, ex vivo analysis of REIMS lipid profiles from 27 patients undergoing STS surgery demonstrated 95.5% accuracy in differentiating non-WDLPS STS from normal tissues and 98.3% accuracy in classifying non-WDLPS STS, adipose, and muscle tissues. While WDLPS could not be distinguished from adipose tissue, leiomyosarcomas were identified with 96.6% accuracy. In two in vivo experiments, an intense spectrometric signal could be obtained from tissue resection within a few seconds, demonstrating the ability to assist with tumor recognition and resection in real-time [77].

### 3.4. Optical Coherence Tomography (OCT)

Mesa et al. explored optical coherence tomography (OCT), a high-resolution imaging technique using near-infrared light, for intraoperative sarcoma margin assessment. In 19 veterinary sarcoma surgeries, OCT images were analyzed with a texture-based algorithm to differentiate tumor, muscle, and adipose tissue. While adipose tissue was easily distinguished, tumor and muscle required statistical metrics for improved classification. Their findings support the use of OCT as a potential real-time tool for improving surgical margin delineation in sarcoma resection [38].

### 3.5. Intraoperative Ultrasound

Intraoperative ultrasonography is well-established as a tool for the detection and resection of liver masses, such as hepatocellular carcinoma and hepatic metastatic disease, and of renal tumors, such as renal cell carcinoma [78,79,80]. It has also been explored for per-operative localization of breast cancer and select pancreatic masses [81,82]. Few studies have presented data in favor of its use in small, non-palpable soft tissue tumors [83,84,85].

Takeuchi et al. evaluated IOUS for non-palpable, ill-defined, or fascia-adjacent small STS (mean tumor size of 5.1 cm) in 19 patients, achieving R0 margins in 18 cases. Two patients experienced local recurrence, resulting in a 5-year recurrence-free survival of 88.9% [85]. Similarly, Farfalli et al. assessed the ultrasound-assisted excision of 22 small tumors (<3 cm) in the deep panniculus, fascia, or muscle, achieving accurate localization in all cases [84]. The procedure allowed for the identification of three additional nodules, which were not diagnosed pre-operatively. All tumors were excised with R0 margins. Giannotti et al. further demonstrated IOUS utility in two cases of non-palpable abdominal wall recurrences, one liposarcoma and one desmoid tumor, which were both successfully localized and resected [86].

Studies highlight IOUS as a valuable, cost-effective tool for precise localization and excision of small, non-palpable soft tissue tumors. However, its limitations include difficulty in delineating infiltrative tumors (e.g., undifferentiated pleomorphic sarcomas, myxofibrosarcomas), large STS often found in the retroperitoneum, its operator-dependent nature requiring experience and a learning curve for optimal use, and its inability to detect microscopic tumor cells, as ultrasound resolution is limited to the macroscopic level [84,85,86].

### 3.6. Artificial Intelligence, Augmented Reality, and Future Multimodal Approaches

Although no artificial intelligence (AI) or augmented reality (AR) platform has yet been validated for *intraoperative* margin assessment in soft tissue sarcoma, AI-assisted digital pathology is emerging as a promising field. In a recent study, Humbert et al. developed and validated a deep learning model applied to digitized histopathology slides of 308 soft tissue sarcoma patients, and showed that a model based on tumor center + periphery (but not margin tissue) predicts metastatic relapse-free survival more accurately than the standard FNCLCC grading system [87]. This study is mentioned for context, but was not included in our analysis, as it does not evaluate intraoperative margin assessment. Nevertheless, it demonstrates how computational analysis could ultimately enhance future AI/AR multimodal platforms for real-time intraoperative guidance in STS surgery.

The main findings from the most relevant studies for these modalities are synthesized in Table 1.

## 4. Discussion

Resection margin status represents a significant prognostic factor following STS resection [22,23,24,25,26,27,28]. Currently available techniques, such as frozen sections, present significant shortcomings that limit their clinical use. Introducing new methods allowing for accurate per-operative evaluation of resection margin status could improve patient outcomes.

This review presents some limitations. The presented studies were heterogeneous regarding their population, scope, level of clinical integration of the proposed technique, and selection of outcome evaluation. This made the comparison between these techniques difficult and inherently subjective. Furthermore, the searched databases were healthcare-centered and would not include techniques that have been published in other relevant domains of the literature, such as engineering journals. This limited the scope of the review, resulting in potentially overlooking other techniques in earlier phases of their development. We also purposefully focused our study on sarcoma and did not include technologies studied on other tumoral sites. Hence, this might have restricted the number of publications reviewed, albeit respecting our inclusion criteria on STS.

Beyond technical performance, successful use of these modalities requires integration into the multidisciplinary surgical workflow. Real-time tools must integrate easily into established clinical workflows and available resources. Several practical barriers also limit broader adoption, including the need for specialized equipment, additional training, and the challenge of incorporating these techniques without disrupting operative flow [41,42,43,44,46,47,48,49,50,60,61,88]. Moreover, cost considerations and the lack of large prospective validation studies further restrict broad clinical use.

In this review, multiple techniques have been evaluated for intraoperative margin assessment in STS, comparing clinical applicability, efficacy, and accessibility. Each technique offers distinct advantages and limitations (Table 2). Among fluorescence-guided modalities, ICG has the most clinical support. Prospective data from Brookes et al. demonstrate a significant reduction in unexpected positive margins (5.1% vs. 25%) when ICG was incorporated for surgical decision making [49]. Its advantages include real-time visualization, an established safety profile, and widespread availability of near-infrared imaging systems [33,49]. Comparatively, acridine orange demonstrates high specificity for sarcoma cells and reduced local recurrence in small case series, suggesting potential benefit, but remains experimental with the limited large-scale clinical validation and lack of data regarding long-term safety [54,55,56].

Within spectroscopy-based techniques, Raman spectroscopy is particularly promising. It has been shown to achieve >90% accuracy in identifying most common subtypes, while providing molecular-level tissue characterization [40,41]. The development of high-speed handheld probes renders intraoperative integration feasible, addressing previous concerns about prolonged acquisition times [41]. Its speed, accuracy, and high level of information make it a promising real-time margin assessment tool for STS. Rapid Evaporative Ionization Mass Spectrometry (REIMS) and diffuse reflectance spectroscopy (DRS) both show early promise with high accuracy (>90%), but remain experimental and require further validation before undergoing clinical studies [74,75,76,77]. IOUS can provide reliable guidance for small, non-palpable tumors, but is limited to macroscopic tumor detection and operator-dependent performance [85].

Our secondary objective was to evaluate the potential impact of these techniques on margin status and oncologic outcomes. Among the reviewed modalities, ICG-guided surgery was the only technique associated with a significant decrease in unexpected positive margins (5.1% vs. 25%) [49]. AO-assisted resection showed reduced local recurrence in small series, suggesting potential benefit in carefully selected cases [55,56,57,58]. In contrast, none of the spectroscopy-based or ultrasound-based technologies have been studied in clinical trials powered to evaluate recurrence, survival, or other oncologic endpoints. Despite these techniques demonstrating excellent diagnostic or visualization capabilities, long-term data on oncological outcomes are lacking [39,89].

To date, the clinical evidence suggests that no single technology can provide the ideal margin evaluation of all subtypes of STS. Designing a multimodal approach tailored to tumor location, histological subtype, and surgical planning may be the best strategy for improving margin assessment. So far, ICG fluorescence guidance represents the most promising and versatile technology to use with various STS clinical presentations.

## 5. Conclusions

Achieving optimal margin status in STS surgery remains a critical determinant of patient outcomes. The integration of emerging intraoperative margin evaluation methods in STS surgery represents a shift from subjective surgical assessment to objective, real-time imaging or molecular guidance. Future studies should integrate multimodal approaches and validate emerging techniques to establish standardized margin-assessment protocols.

## Figures and Tables

**Table 1:** Summary of important publications describing intraoperative STS margin identification techniques.

Author	Publication Year	Technique	Key Results
Nicoli et al. [37]	2021	ICG Fluorescence	9/11 (81.8%) of STS demonstrated significant fluorescence No adverse events
Brookes et al. [49]	2021	ICG Fluorescence	37/39 (94.9%) of STS demonstrated significant fluorescence 11/39 (28.2%) of resections using ICG were guided by fluorescence Unexpected positive margin risk of 5.1% in group using ICG fluorescence vs. 25.0% in standard surgery group
Gong et al. [50]	2023	ICG Fluorescence	Demonstration of technique’s high specificity (89%) and low sensitivity (22.2%) Correlation of margins assessment with final pathology assessment in 56% of cases
Matsubara et al. [58]	2013	Acridine Orange	Comparable local recurrence risk between marginal resection + AO group vs. conventional resection group Comparable 10-year OS between marginal resection + AO group vs. conventional resection group
Tsuchie et al. [56]	2019	Acridine Orange	Reduction in risk of local recurrence in marginal resection + AO group vs. wide resection group (33.3% vs. 47.1%).
Nguyen et al. [39]	2017	Raman spectroscopy	Sensitivity of 59.6% and specificity of 91.6% in detecting STS Inability to detect WDLPS: improvement of sensitivity to 89.5% and specificity to 96.4% after excluding WDLPS
Dulude et al. [41]	2025	Raman spectroscopy	Sensitivity of 94%, specificity of 95%, and accuracy of 94% when differentiating WDLPS from adipose tissue Sensitivity, specificity, and accuracy > 90% in differentiating liposarcomas from adipose tissue, and >80% in differentiating non-liposarcoma STS from non-adipose tissue
Geldof et al. [72]	2024	Diffuse reflectance spectroscopy	Ex vivo classification accuracy of 90%, sensitivity of 88%, and specificity of 93% in identifying STS (including WDLPS)
Vaysse et al. [77]	2024	Rapid Evaporative Ionization Mass Spectrometry	98.3% accuracy in classifying non-WDLPS STS from adipose and muscle tissues 96.6% accuracy in identifying leiomyosarcoma WDLPS could not be differentiated from adipose tissue
Mesa et al. [38]	2017	Optical Coherence Tomography	In animal studies, ability to correctly identify STS, adipose, and muscle tissue using statistical analysis
Farfalli et al. [84]	2011	Intraoperative ultrasound	100% R0 resections using IOUS 3/22 (13.6%) cases had additional sites of disease identified using IOUS
Takeuchi et al. [85]	2020	Intraoperative ultrasound	18/19 (94.7%) R0 resection using IOUS 5-year RFS of 88.9%

**Table 2:** Various intraoperative margin assessment techniques for STS.

Method	Technique/Principle	Main Advantages	Main Limitations
Frozen Section	Intraoperative cryosection of margins to assess residual tumor	Gold standard for final pathology Widely used	Not always reliable in STS Falsely reassuring Adds cost Minimal impact on outcomes
ICG Fluorescence	NIR dye uptake by tumors via EPR effect and clarithrin-mediated endocytosis.	Real-time visualization Safe Available Reduced positive margins (5.1% vs. 25%).	Background signal Variable uptake Non-standardized dosing Low sensitivity in necrotic tumors
Acridine Orange (AO)	Fluorescent dye accumulates in sarcoma cells Activated by light/radiation	Reduces local recurrence Preserves function Comparable to wide excision No major short-term toxicity	Experimental Not FDA-approved Needs special equipment Possible genotoxicity Limited validation.
Raman Spectroscopy	Detects molecular vibrations for tissue biochemical fingerprinting.	>90% accuracy (UltraProbe) Real-time visualization Molecular specificity No dye needed	Subtype-specific algorithms needed Experimental
Diffuse Reflectance (DRS)	Measures reflected broadband light to analyze tissue composition.	Simple, fast, real-time; accuracy ~90 No dye needed.	Small sample Ex vivo only Low spatial/biochemical resolution
REIMS	Analyzes electrosurgical vapors for real-time molecular profiles.	>95% accuracy; real-time second Distinguishes sarcoma vs. normal	Limited to pilot data Cannot separate WDLPS from fat Destructive technique
OCT	NIR imaging for microstructural differentiation.	Real-time Distinguishes adipose vs. tumor Depth-resolved information Promising pilot data	Structural imaging modality Needs texture analysis; only preclinical data.
Intraoperative Ultrasound (IOUS)	Real-time ultrasound localization during surgery.	Accurate for small/impalpable tumors High R0 resection Low cost	Operator-dependent Limited for large/infiltrative STS Macroscopic only

## Data Availability

No new data were created or analyzed in this study. Data sharing is not applicable to this article.
